# A Web-Based, Hospital-Wide Health Care-Associated Bloodstream Infection Surveillance and Classification System: Development and Evaluation

**DOI:** 10.2196/medinform.4171

**Published:** 2015-09-21

**Authors:** Yi-Ju Tseng, Jung-Hsuan Wu, Hui-Chi Lin, Ming-Yuan Chen, Xiao-Ou Ping, Chun-Chuan Sun, Rung-Ji Shang, Wang-Huei Sheng, Yee-Chun Chen, Feipei Lai, Shan-Chwen Chang

**Affiliations:** ^1^ Graduate Institute of Biomedical Electronics and Bioinformatics National Taiwan University Taipei Taiwan; ^2^ Computational Health Informatics Program Boston Children’s Hospital Boston, MA United States; ^3^ Department of Electrical Engineering National Taiwan University Taipei Taiwan; ^4^ Center for Infection Control National Taiwan University Hospital Taipei Taiwan; ^5^ Information Systems Office National Taiwan University Hospital Taipei Taiwan; ^6^ Department of Computer Science and Information Engineering National Taiwan University Taipei Taiwan; ^7^ Department of Internal Medicine National Taiwan University Hospital and College of Medicine Taipei Taiwan; ^8^ National Institute of Infectious Diseases and Vaccinology National Health Research Institutes Miaoli Taiwan

**Keywords:** health care-associated infection, infection control, information systems, surveillance, Web-based services

## Abstract

**Background:**

Surveillance of health care-associated infections is an essential component of infection prevention programs, but conventional systems are labor intensive and performance dependent.

**Objective:**

To develop an automatic surveillance and classification system for health care-associated bloodstream infection (HABSI), and to evaluate its performance by comparing it with a conventional infection control personnel (ICP)-based surveillance system.

**Methods:**

We developed a Web-based system that was integrated into the medical information system of a 2200-bed teaching hospital in Taiwan. The system automatically detects and classifies HABSIs.

**Results:**

In this study, the number of computer-detected HABSIs correlated closely with the number of HABSIs detected by ICP by department (n=20; *r*=.999 *P*<.001) and by time (n=14; *r*=.941; *P*<.001). Compared with reference standards, this system performed excellently with regard to sensitivity (98.16%), specificity (99.96%), positive predictive value (95.81%), and negative predictive value (99.98%). The system enabled decreasing the delay in confirmation of HABSI cases, on average, by 29 days.

**Conclusions:**

This system provides reliable and objective HABSI data for quality indicators, improving the delay caused by a conventional surveillance system.

## Introduction

### Background

Health care-associated infections (HAIs), adverse events related to health care, excess mortality and morbidity, and resource use are responsible for augmenting antimicrobial resistance [1,2]. Surveillance of HAIs is an essential component of infection control programs in health care settings. The goals of surveillance are to assess the disease incidence, identify the niche and opportunity for improvement, monitor the efficacy of interventions, and support the rationale behind changes in policies [3]. Previous studies have reported that HAIs have decreased ly in hospitals that adopted surveillance programs in the 1980s in the United States [4,5]. In 1981, a hospital-wide HAI surveillance program was initiated at the National Taiwan University Hospital (NTUH). Factors such as periodic feedback to the departments or wards and intensified interventions resulted in a decrease in surgical-site and respiratory tract infections in the 1980s [6]. Following the upgrading of the infection prevention and control program in 2004, there was a significant reduction in bloodstream infections, HAIs in intensive care units, and HAIs caused by multidrug-resistant organisms (MDROs) during the period from 2004 to 2007 [7].

Attention to HAIs has increased partially because of legislative mandates for reporting and reimbursement policies [8]. However, conventional HAI surveillance systems require considerable human involvement in integrating and interpreting data and are labor intensive, performance dependent, and tend to divert resources that are necessary for implementing control measures and prevention activities [9]. Relying on employees in institutions in an environment where reporting HAIs can be associated with punitive consequences is suboptimal [10]. Furthermore, the decision rules (ie, the case definitions) of HAIs are complicated when the complexity of the current health care in tertiary care hospitals is considered. Utilization of hospital discharge registry data delays the detection of HAI, eventually resulting in ineffective identification of problems [11]. Recent studies have identified interinstitutional variability in surveillance techniques, an inconsistency that affects the validity of publicly reported HAI data [12]. Developing reliable and objective HAI definitions and automated processes for infection detection is crucial; however, transformation into automated surveillance system remains challenging [8,10].

### Study Objective

In continuation with our previous efforts in developing a Web-based MDRO surveillance system that automatically identifies and accurately detects suspicious outbreaks of MDROs [13], implementation of which could save 1 person-day daily, we conducted this study with the aim of developing a Web-based automatic surveillance and classification system for health care-associated bloodstream infection (HABSI), the most common HAIs at NTUH [6]. In addition, performance of the system was evaluated by comparing the proposed system with a conventional infection control personnel (ICP)-based surveillance system.

## Methods

### Hospital Setting and Study Population

The study was conducted at NTUH, a 2200-bed teaching hospital that provides primary and tertiary care for the adult and the pediatric population in Taiwan. This study was approved by the Institutional Ethics Review Board of NTUH (NTUH-200904014R). In 2011, NTUH served 2,309,108 outpatients, received 106,950 emergency visits, and discharged 104,899 patients (723,505 patient-days).

Two sets of blood samples from separate venipuncture sites for bacterial culture were routinely collected from patients who were newly diagnosed with sepsis. An additional sample was collected after 45-60 minutes to define continuous bloodstream infection (BSI). Only 1 blood sample for a follow-up culture was collected to confirm the clearance of BSI. Of the 80,327 blood specimens that were sent for isolation and identification of pathogens, 991 (1.23%) were obtained through a single blood draw. A total of 8745 samples grew 1 or more pathogens (10.88%); of these, 1908 exhibited HABSIs. The pooled mean of HABSI incidence was 14.7 episodes/1000 patients (range 0.2-112.7/1000 patients by department) and 2.13 episodes/1000 patient-days.

### Conventional ICP-Based HAI Surveillance System

A prospective, hospital-wide on-site surveillance of HAIs, initiated in 1981 [6], was conducted by ICPs who reviewed microbiological data daily and visited inpatient units weekly to identify patients with HAIs according to definitions of the Centers for Disease Control and Prevention (CDC) [14] before we implemented the Web-based surveillance system. If required, the ICPs consulted physicians, particularly infectious disease physicians, to interpret the medical information of patients who have received complicated interventions. Data are collected on standardized data-collection forms and input into the computer database manually. In addition, the ICPs monitor culture results from the clinical microbiology laboratory daily to identify MDROs. The unit-specific incidences of HAIs, including overall, unit-based, and site-specific infection rates, are analyzed monthly and compared with historical data. Feedback is provided to each unit to stimulate intervention measures.

The key data are collected by systematically reviewing hospital information systems (HISs), laboratory information systems (LISs), and handwritten charts. Because of advances in medical information system, data of HISs and LISs are currently stored as electronic medical records (EMRs). However, data generated in the medical information system are scattered in numerous databases, and data access is hindered by several interfaces. In addition, data must be integrated, interpreted, and transformed into meaningful information.

### Web-Based HAI Surveillance and Classification System

**Figure 1:**
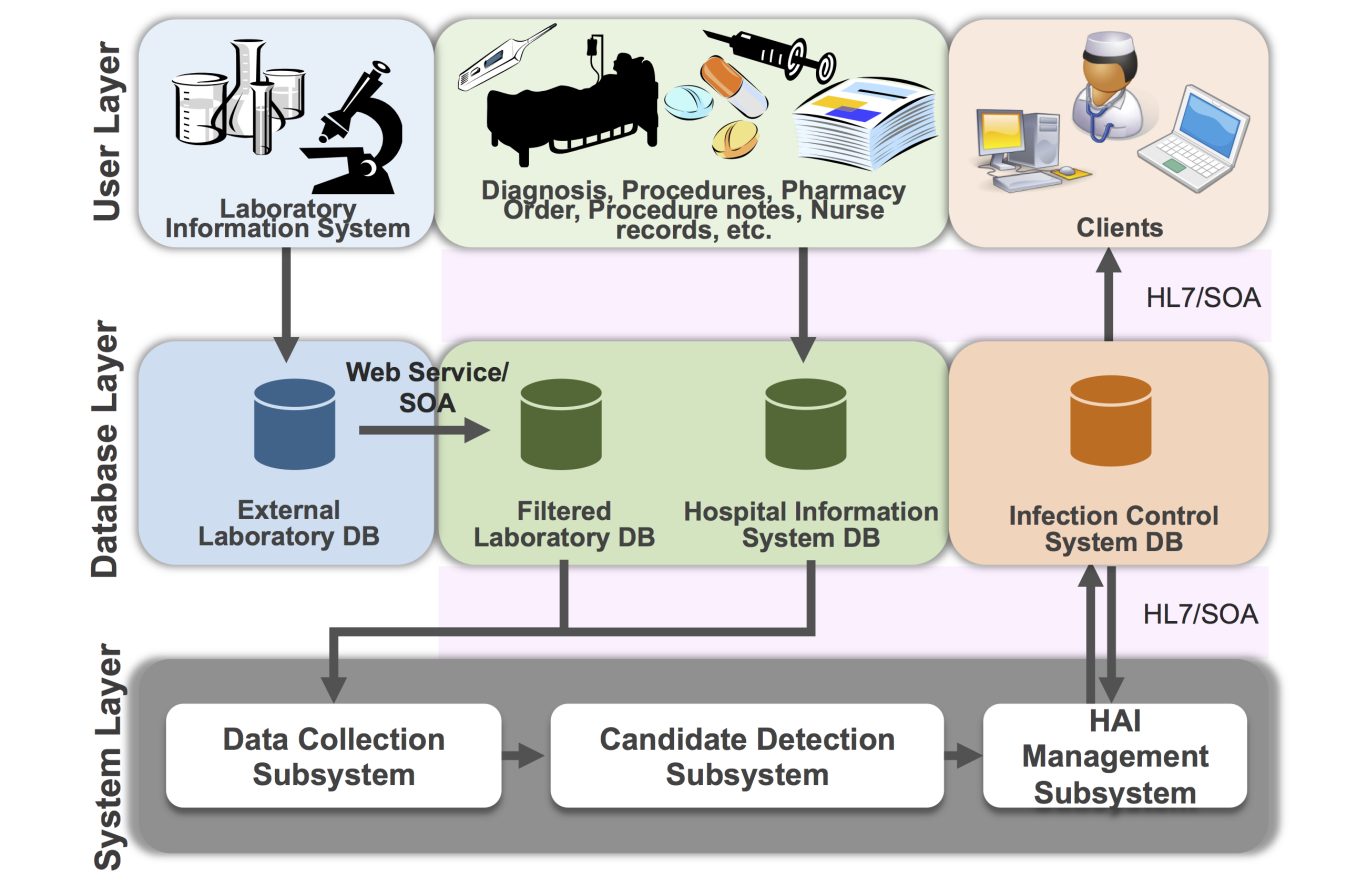
General architecture of the Web-based health care-associated infection (HAI) surveillance and classification system. DB: database; HL7: Health Level Seven; SOA: service-oriented architecture.

We established a rule-based HABSI surveillance and classification system (the system), which was implemented on October 1, 2010. The current version was revised on September 20, 2012 (Figure 1). The system screens HIS and LIS data daily to detect HABSI candidates according to the well-defined detection rules. The system detects and classifies HABSIs automatically and reserves professional autonomy by requiring further confirmation. Figure 1 shows the general architecture of the system, including the user layer, the database layer, and the 3-part system layer (data collection, candidate detection, and HAI management). The system adheres to service-oriented architecture (SOA) and Health Level Seven (HL7) standards and can be adapted in other information systems [15].

This data-collection subsystem collects relevant data from HIS and LIS using HL7 standards, which was extended stepwise from the previous version [13]. For example, data related to age, sex, ward transfer, admission date, and discharge diagnosis are obtained from the hospital administrative system. Re-admissions within 2 days of discharge are linked to the previous hospital stay and considered to be a single hospitalization episode. Procedure codes are obtained from the hospital billing system. Data on body temperature, heart rate, respiratory rate, and presence of device including indwelling catheters are obtained from the nursing system. The use of antimicrobial agents is obtained from the pharmacology prescription system. The laboratory data consist of specimen information and microbiological data. We used the specimen log-in time as a proxy for the time of specimen collection at bedside and the infection time.

**Figure 2:**
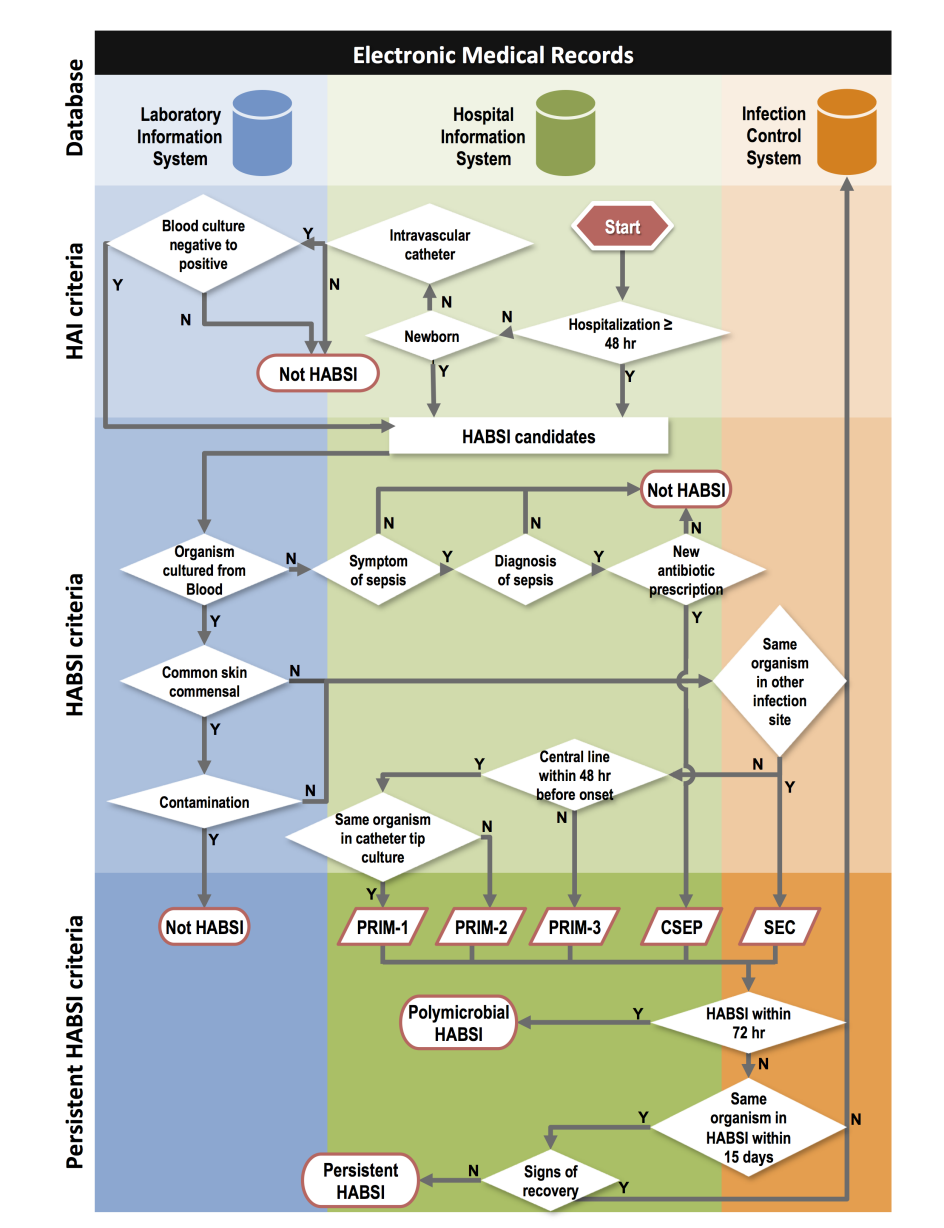
Computer algorithms to detect health care-associated bloodstream infection (HABSI) by active daily screening of data from hospital information system and laboratory information system. HABSIs are classified into primary HABSI (PRIM), secondary HABSI (SEC), and clinical sepsis (CSEP) as described in Multimedia Appendix 1. Polymicrobial and persistent BSI criterion here are to eliminate false signals due to duplicate counting, etc.

To develop the detection rules in the candidate-detection subsystem, the ICPs have adapted objective components of the National Health Care Safety Network (NHSN) definitions established by the CDC [14] and modified them according to the consensus between Taiwan Center for Disease Control and NTUH (see Multimedia Appendix 1 for detection rules and Multimedia Appendices 2 and 3 for list of devices and signs and symptoms, respectively). Computer engineers have established HABSI detection rules accordingly (Figure 2). Thus, there are differences between the detection rules and NHSN definition. Although the secondary HABSI was removed from the NHSN definition in 2008, we used the data in the NTUH infection control system to maintain data consistency for time trend analysis [6,7]. The primary HABSI was divided into 3 subtypes for quality-improvement purpose, including central line-related BSI (CRBSI), central line-associated BSI (other than CRBSI), and other primary HABSI. Detection rules did not include free text data that were not available in the EMRs, such as chills and apnea, during the study period.

The HAI management subsystem, established on July 1, 2007 [13], and upgraded periodically (refer to the screenshot in Multimedia Appendix 4), consists of data integration, display, and a data-modification user interface for facilitating the surveillance of HAIs. The HAI management subsystem has a single entry point for HIS through the browser [16] and comprises a storage information component relevant to HAI. The information for each event of HAI includes patient demographic data, diagnoses, procedures, medications, and microbiology reports to facilitate the confirmation of HAI by ICPs. If required, the ICPs can modify these HABSI cases or add additional HABSIs that were undetectable by the subsystem. The latter might occur for new units, new pathogens, new procedures, or other elements, which are not yet included in the current database. Furthermore, the system provides data analysis and process-control charts [13].

### Evaluation of System Performance and Statistical Analyses

**Figure 3:**
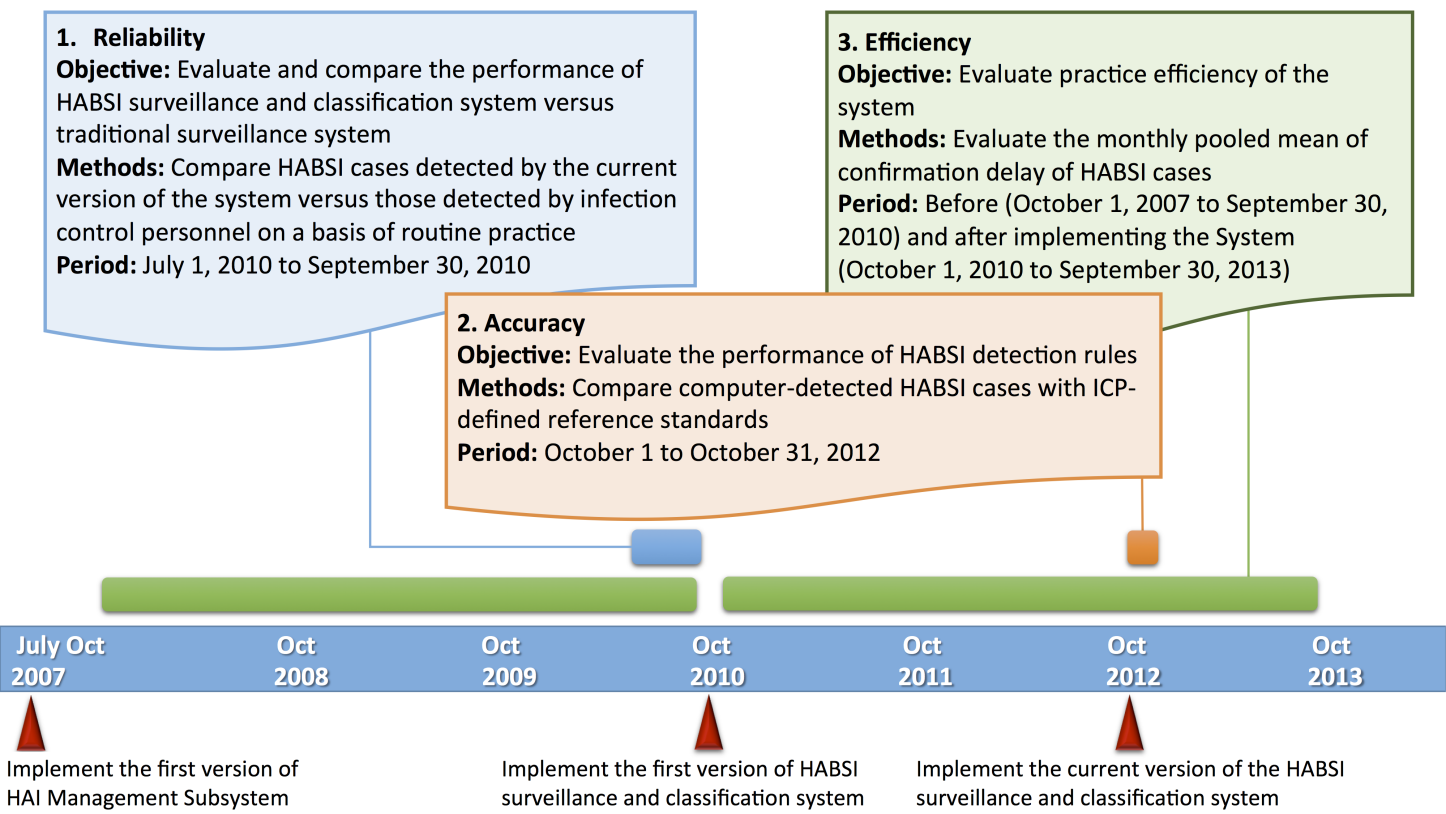
Timeline of development and performance evaluation of the health care-associated bloodstream infections (HABSIs) surveillance and classification system. ICP: infection control personnel.

Clinically useful tests must be valid and reliable and have a reasonable turnaround time. Thus, we conducted a 3-aspect evaluation, including accuracy, reliability, and efficiency, of the system. Figure 3 summarizes the objectives, methods, and evaluation periods. Reliability of the system was evaluated before implementation of the first version of the system in 2010; accuracy of HABSI rules was evaluated in October 2012. Furthermore, we evaluated, and continue to evaluate, the stepwise improvement in efficiency after implementation of the HAI management system and the HABSI surveillance and classification system.

We first evaluated the performance of the system during the developmental phase (ie, before implementation of the system) regarding its potential to provide data for quality indicators. Computer-detected HABSIs were compared with ICP-detected HABSIs as a routine practice between July 1, 2010, and September 30, 2010. The correlation between these 2 data sources was analyzed according to department distribution and time trend of HABSIs.

On the basis of inconsistent and varied performances of the conventional ICP-based surveillance system, we further evaluated the performance after implementing the system using ICP-defined reference standards. To generate high-quality reference standards, 11 ICPs performed a retrospective review of all medical data of patients who were admitted between October 1, 2012, and October 31, 2012, to identify HABSI cases based on NTUH detection rules, and one of the authors (H-CL) validated the results. The sensitivity, specificity, positive predictive value (PPV), negative predictive value (NPV) of the system, and Cohen kappa coefficient [17] were calculated in this evaluation method. All the performance indicators were calculated based on whether a patient had HABSI or not.

We then compared the delay in HABSI confirmation (as a proxy for practice efficiency) before (October 2007-September 2010) and after (October 2010-September 2013) the system implementation. The delay in confirmation was defined as the intervals between the HABSI confirmation dates (complete data entry and confirmed by ICPs in the HAI management subsystem) and log-in dates of the first blood specimen with positive results and was calculated by averaging the number of delay days in each month. The study periods were selected taking into account the seasonal variation of HAI rates demonstrated previously [7].

## Results

### Performance of the System as a Provider of Quality Indicator

**Figure 4:**
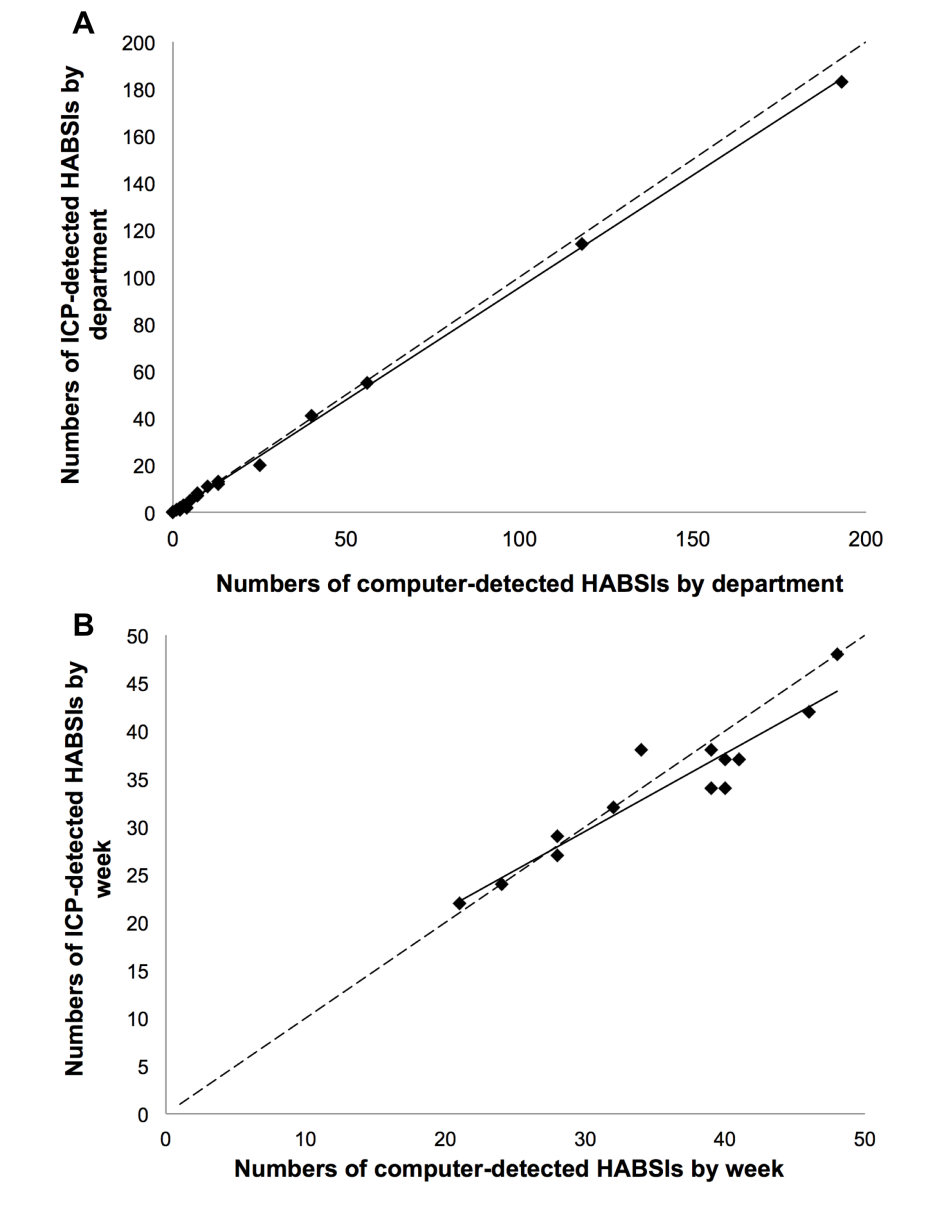
Correlation of 501 episodes of infection control personnel (ICP)-detected health care-associated bloodstream infection (HABSI) and 479 episodes of computer-detected HABSI from 20 departments during the 14-week study period. (A) Perfect agreement of HABSI episodes by department (n=20, Pearson correlation, *r*<.999, *P*<.001). (B) Perfect agreement of HABSI episodes by time (n=14, Pearson correlation, *r*=.941, *P*<.001).

During the 14-week study period (July 1, 2010-September 30, 2010), 501 episodes of ICP-detected HABSIs and 479 episodes of computer-detected HABSIs were found throughout the 20 departments. The data were highly correlated by place and time (Figure 4). These results indicated that the data provided by the system are suitable quality indicators. Thus, we implemented the system on October 1, 2010.

### Accuracy of the Detection Rule

**Figure 5:**
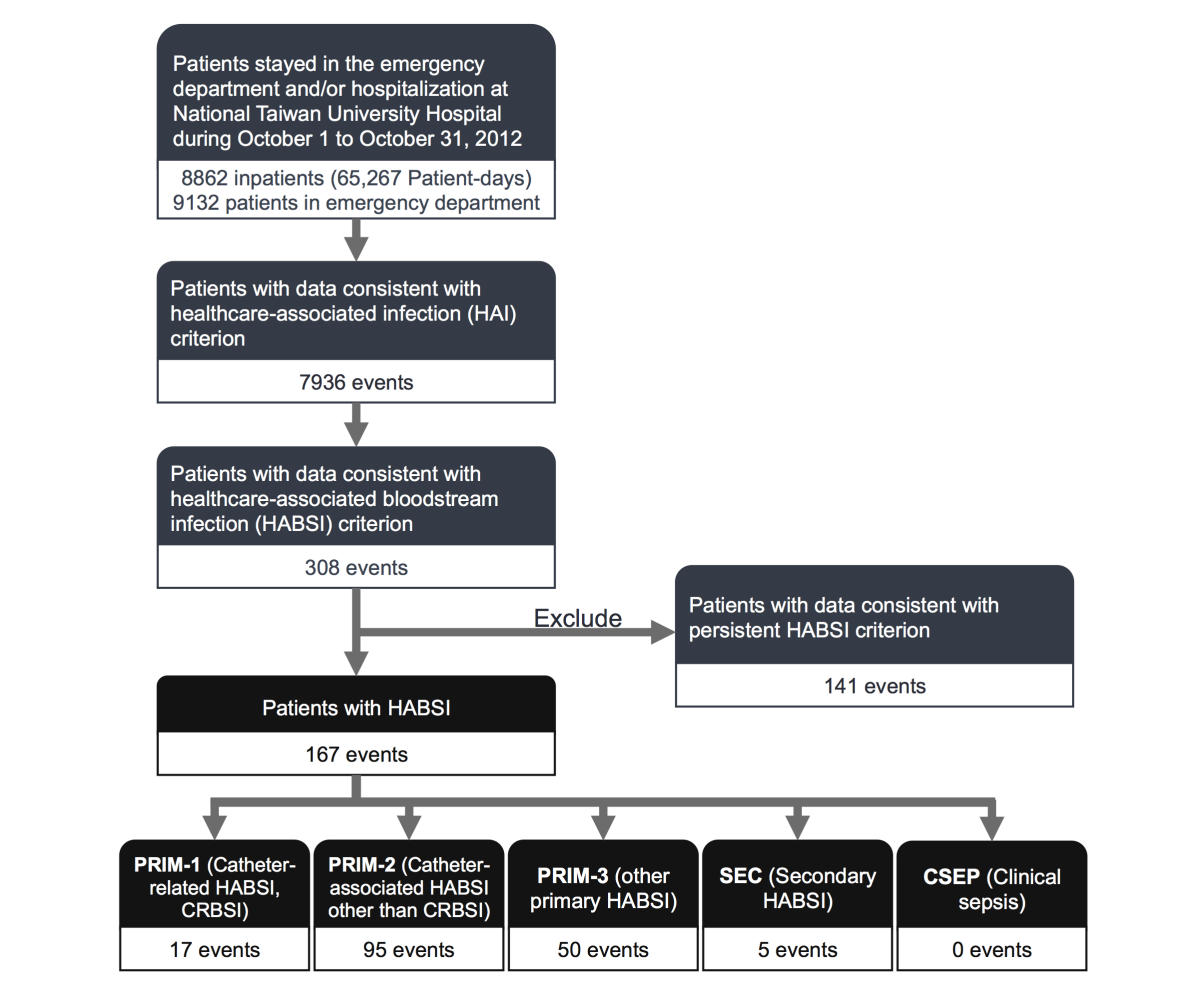
Computer algorithms identifying 167 events of health care-associated bloodstream infection among 8862 inpatients and 9132 patients in the emergency department between October 1 and October 31, 2012 (31 days).

In October 2012, the system identified 167 episodes of HABSIs (Figure 5), including 160 of 163 reference standard episodes in 31 days (Table 1). The sensitivity and specificity of the HABSI classification system were 98.16% (95% CI 94.29-99.52) and 99.96% (95% CI 99.91-99.98), respectively. The PPV and NPV were 95.81% (95% CI 91.22-98.15) and 99.98% (95% CI 99.95-100.00), respectively. Moreover, the agreement between the computer-detected HABSIs and the reference standard was nearly perfect (Cohen kappa coefficient .97; 95% CI 0.95-0.99). The performance of the system for detecting central line-associated HABSI was also excellent (sensitivity 97.14%, specificity 99.94%, PPV 91.07%, NPV 99.94%, and Cohen kappa coefficient .94).

**Table 1:** Comparison of the case detection results of the health care-associated bloodstream infection surveillance and classification system with infection control personnel reference standard between the periods October 1 and October 31, 2012.

Infection control personnel reference standard	Automated surveillance classification		
	HABSI	Not HABSI	Total
HABSI	160	3^a^	163
Not HABSI	7^b^	17,824	17,831
Total	167	17,827	17,994

^a^Retrospective review by 2 investigators independently confirmed that these 3 episodes of HABSI due to common skin commensals were missed due to fever criteria (temperature > 38°C): 1 patient received antipyretic agents, 1 with a and sustained increase in temperature (>1°C) from baseline but less than 38°C, and in the other patient fever was documented only in the progress note and was missed by using this fever criteria.

^b^Four false-positive cases due to revision of final laboratory reports after “recall day.” One episode of community-acquired BSI was detected as HABSI due to delay in transportation of specimen to microbiology laboratory. Two were cases of persistent bloodstream infection.

### Decrease in the Delay of HABSI Confirmation

**Figure 6:**
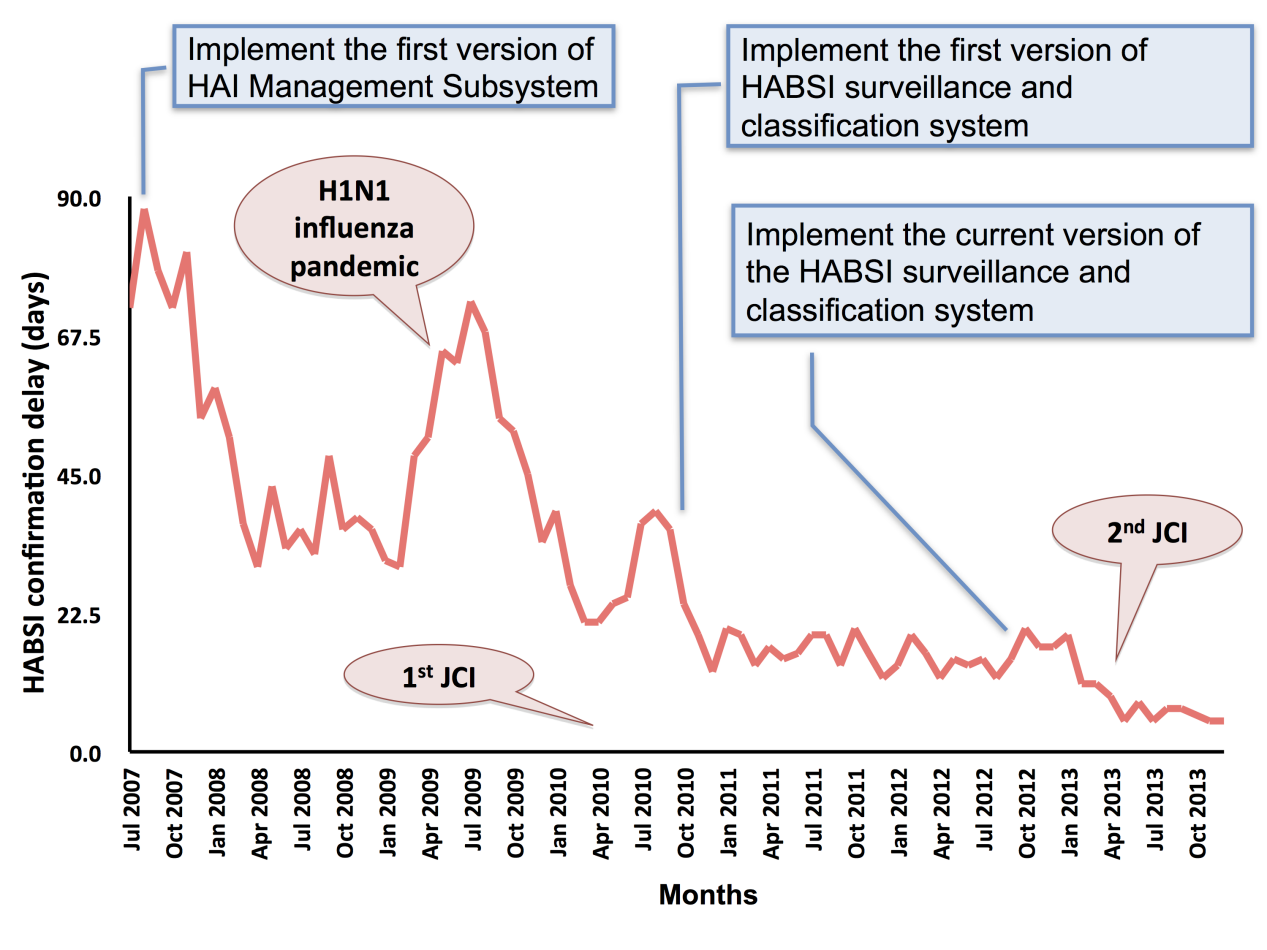
The detection delay of health care-associated bloodstream infection (HABSI) decreased gradually from July 2007 to December 2013. The first version of health care-associated infection (HAI) management subsystem has been developed to facilitate infection control personnel-based surveillance program since July 2007. This was revised stepwise and has been operation as an automatic system since October, 2010. In March 2009, this hospital initiated preparedness for international hospital accreditation, which was scheduled 1 year later. Influenza pandemic occurred in April 2009.

The delay in HABSI confirmation was reduced from 43.58 (SD 15.57) days before the system implementation (October 2007-September 2010, 1096 days) to 14.58 (SD 4.64) days after the implementation (October 2010-September 2013, 1096 days; *P*<.001). Figure 6 shows that the time trend of the delay in HABSI confirmation, which was as high as 90 days in July 2007, decreased after the implementation of the HAI management subsystem in July 2007, and further improved after automating the system in October 2010. The delay in the second half of 2013 was only 5.78 (SD 0.91) days. Conversely, without the system, the delay increased during H1N1 influenza pandemics and when preparing for international accreditation.

## Discussion

### Preliminary Findings

This Web-based, automated HABSI surveillance and classification system used discrete data elements obtained from HISs, and LISs provided data highly correlated with conventional ICP surveillance system. The performance was excellent regarding sensitivity, specificity, PPV, and NPV, and was in agreement with reference standards; the system reduced the delay in confirmation, on average, by 29 days. The system improves practice efficiency, enabling ICPs to intensify intervention and further reduce HAI rates.

Computer-assisted HAI surveillance and classification systems are widely implemented [11,18-35]. Studies have demonstrated using various algorithms for detecting HAIs, although with varied outcomes (summary in Multimedia Appendix 5) [25-35]. The most critical performance characteristics of these kinds of surveillance systems are sensitivity and NPV; the efficiency of the system can be assessed according to the PPV [36]. Compared with reference standards, the current version of detection rules and computer algorithms performed excellently with regard to these 3 parameters (sensitivity 98.16%, NPV 99.98%, and PPV 95.81%) because of the following reasons: Through cross-talk among ICPs, infectious disease physicians, and engineers, we integrated clinical know-how and translated international case definitions to define detection rules and construct computer algorithms. The system was established and revised through a plan-do-check-act cycle in a general hospital, which serves a varied patient population and offers numerous procedures. Furthermore, we evaluated the clinical utility of the system, comparing it with the prospective, hospital-wide, conventional surveillance system and reference standards.

In this study, the HABSI detection rules (see Multimedia Appendix 1) were clearly defined, and computer algorithms (Figure 2) provided excellent results. We adapted the US CDC definition of HAI, adding rules related to re-admission within 48 hours and neonates, and a “hospital-acquired” rule, defined as a positive blood culture that was obtained 48 hours or more after admission. These rules included rules for clinical sepsis, and the system actively screened the heart rate, respiratory rate, and body temperature for infection-related symptoms and signs. These efforts facilitated ameliorating the potential underestimation of HAI when only laboratory data were used. The HABSI detection rules included polymicrobial and persistent BSI criterion to eliminate the majority of false signals (eg, duplicate counting). In addition, classification of HABSIs is flexible to addressing local policy and ICP requests to compare them with the NTUH historical data.

The system detects and classifies HABSIs automatically and ensures professional autonomy by requiring further confirmation. Each episode of HAI requires confirmation by ICPs. The system presents detailed information about each HABSI candidate systematically to support decision making. The main reason for this design is because the system is imperfect (see the “Limitations” section). Furthermore, because HAIs are rare in hospitalized patients, the system aims to select potential HAI candidates and exclude patients who do not have an HAI and hence do not require review by ICPs.

This study verifies the potential of the system to provide data for quality indicators. The system enabled sustainable surveillance, generating data that were correlated with conventional surveillance systems by department and time. In addition, the delay in HABSI confirmation decreased to 5.78 (SD 0.91) days in the second half of 2013. Because of the reduced length of hospital stay and the increased threat of emerging infectious diseases, early detection of HAIs can enable identifying the reservoir or index case and providing early intervention before pathogens spread further. Currently, the delay in HABSI confirmation is caused by the time required to identify the positive blood cultures and microorganisms; the system detects and analyzes results of blood cultures to prevent false alerts. Furthermore, the major challenge encountered when sharing automated HABSI surveillance systems between hospitals is different HIS settings [22]; the Web-based system, which adheres to SOA and HL7 standards, can be easily extended to and adapted for use with other medical information systems.

### Limitations

Although our results suggested that the system performs well, this study had several limitations. First, data integrity and instantaneity substantially affect performance, because this system uses EMRs from many sources. In addition, not all data required for HAI surveillance [37] are available on the Web, because EMRs were not fully implemented at NTUH until January 1, 2014. Second, the quality of source data, which is related to the performance of clinical practice and EMRs, affected the results. As much as 1.2% (991/80,327) of the blood specimens collected in 2011 were obtained through a single blood draw (reasons described in the “Methods” section), and this affected the identification of common skin commensals and resulted in a false-positive HABSI case (Table 1). We reviewed the medical records and determined that the false-positive result was caused by the delay in specimen delivery and log-in time. Third, the system updates laboratory data from LIS within a fixed period (recall day); however, data are not repeated during a subsequent hospital stay. This resulted in 4 false-positive HABSI cases, because the laboratory reports were revised in the LIS after recall day and included common skin commensal contaminants that were not updated in the system.

Fourth, the agreement regarding the place of onset (responsible ward) was not evaluated in this study, because patients are frequently transferred to different wards and electronic clinical data regarding symptoms and signs of infection were unavailable during the study period. Fifth, the case definition of HABSI is complicated when clinical scenarios are taken into consideration. For disagreements and received revaluations (Table 1), all the false-negative results were due to the case definition of fever (>38°C). Furthermore, we did not evaluate the reduction of person-hours after implementing the system, as described in previous studies [13,38,39], or the subsequent effect on the reduction of HABSI by reallocating ICP time and effort from collecting data to improving program quality, as described in a previous research of a hospital-wide hand-hygiene promotion program [7] and care bundles for device-associated infection to prevent HABSI.

### Conclusions

This fully automated system that can be integrated in medical information systems detects and classifies HABSI within 5.78 (SD 0.91) days after occurrence, enabling the opportunity for early intervention. Currently, the system and other components of the infection control system [13,39] operate well and have become indispensable tools for infection control programs. Future studies using clinical data from complete EMRs and refining classification algorithms or adopting multivariable prediction models are warranted [36]. According to the results of this pilot study for HABSI automated surveillance, further efforts for other HAI surveillance are underway.

## Appendix

Multimedia Appendix 1 Detection rules for the health care-associated bloodstream infection (HABSI) Surveillance and Classification System at National Taiwan University Hospital (NTUH) and corresponding Centers for Disease Control and Prevention (CDC) National Healthcare Safety Network (NHSN) definition of health care-associated infection (HAI) [14].

Multimedia Appendix 2 The list of central lines and intravascular devices.

Multimedia Appendix 3 The definition of symptoms/signs of sepsis in pediatric patients.

Multimedia Appendix 4 Summary of characteristics and performances of computer-assisted health care-associated infection surveillance systems in the literature.

Multimedia Appendix 5 User interface of HAI Management System. This system integrated all the information which was needed for HAI decision.

## Abbreviations

CABSI: central line-associated bloodstream infection
CDC: Disease Control and Prevention
CRBSI: central line-related bloodstream infection
EMRs: electronic medical records
HABSI: health care-associated bloodstream infection
HAIs: health care-associated infections
HIS: hospital information systems
HL7: Health Level Seven
ICP: infection control personnel
LISs: laboratory information systems
MDROs: multidrug-resistant organism
NHSN: National Health Care Safety Network
NPV: negative predictive value
NTUH: National Taiwan University Hospital
PPV: positive predictive value
SOA: service-oriented architecture
