# Supplementary material for: A Web-Based, Hospital-Wide Health Care-Associated Bloodstream Infection Surveillance and Classification System: Development and Evaluation
Source: JMIR Med Inform. 2015 Sep 21;3(3):e31. doi: 10.2196/medinform.4171 (PMC4705006; doi:10.2196/medinform.4171)
Supplement: Multimedia Appendix 1 [file medinform_v3i3e31_app1.pdf]

Criteria	CDC NHSN definition	NTUH detection rules
HAI criterion	A localized or systemic condition resulting from an adverse reaction to the presence of an infectious agent(s) or its toxin(s). There must be no evidence that the infection was present or incubating at the time of admission to the acute care setting.<br><br>The following are not considered health care-associated infections:<br><br>1. Infections associated with complications or extensions of infections already present on admission, unless a change in pathogen or symptoms strongly suggests the acquisition of a new infection.<br><br>2. Infections in infants that have been acquired transplacentally and become evident ≤48 hours after birth.<br><br>3. Reactivation of a latent infection.	Any hospitalized patient who meet at least one of the following criteria to be considered health care-associated infection candidates:<br><br>1. Patient stays in hospital ≥ 48 hours.<br><br>2. Newborn.<br><br>3. Patient has indwelling intravascular catheter[a] and there is a negative culture result or different isolates before positive one.
**HABSI criterion**	*Laboratory-confirmed bloodstream infection 1*: Patient has a pathogen cultured from 1 or more blood specimen and organism cultured from blood is not related to an infection at another site. In addition, the pathogen does not include organisms considered common skin contaminants.<br><br>*Laboratory-confirmed bloodstream infection 2*<br><br>Patient has a common skin	*Patient meet all the following 4 criteria:*<br><br>1. HAI criterion<br><br>2. Organism recovered from 1 or more blood specimen<br><br>3. Not secondary BSI (not meet “SEC” criteria)<br><br>4. Not contamination (common skin commensal[b] recovered from only 1 blood culture or no symptoms/signs[c])

contaminant cultured from 2 or more blood cultures drawn on separate occasions and organism cultured from blood are not related to an infection at another site.

Patient has at least 1 of the following signs or symptoms:

1. ≤1 year of age: fever (>38°C, rectal), hypothermia (<37°C, rectal), apnea, or bradycardia and symptoms.[d]
2. Other age group: fever (>38°C), chills, or hypotension[d]

PRIM-1 (central line-related BSI, CRBSI)

Patient meet all 3 criteria:

1. HABSI
2. Central lines[a] in place within the 48-hour period before first positive blood culture log-in time
3. Organism recovered from blood also recovered from central line tip culture with at least 15 colony forming units within 48 hours after the first positive blood culture log-in time.

PRIM-2 (Central line-associated BSI, CABSI other than CRBSI)

Patient meet all 3 criteria:

1. HABSI
2. Central lines[a] in place within the 48-hour period before first positive blood culture log-in time.
3. Not meet PRIM-1 criteria

PRIM-3 (other primary HABSI)

Patient meet all 3 criteria:

1. HABSI
2. Not meet PRIM-1 criteria
3. Not meet PRIM-2 criteria

Clinical sepsis

CSEP may be used only to report primary BSI in neonates and infants.

Clinical sepsis must meet the

CSEP (Clinical sepsis)

Patient ≤1 year of age has one of the symptoms of sepsis[c] and diagnosis of sepsis[e] and has new antibiotic prescription (before or after symptom

	following criterion:<br><br>Patient ≤1 year of age has at least one of the following clinical signs or symptoms with no other recognized cause: fever (>38°C rectal), hypothermia (<37°C rectal), apnea, or bradycardia and blood culture not done or no organisms detected in blood and no apparent infection at another site and physician institutes treatment for sepsis.	of sepsis within 48 hours).
**Secondary HABSI criterion**		**SEC (Secondary HABSI)**[f]<br><br>Patient meet all 3 criteria:<br><br>1. HAI criterion<br>2. Organism recovered from 1 or more blood specimen<br>3. Organism recovered from blood also recovered from nonblood cultures within 168 hours[g] of first positive blood culture log-in time.
**Polymicrobial HABSI criterion**		Patient meet both criteria:<br><br>1. HAI criterion<br>2. A single blood culture grows multiple organisms.<br><br>OR<br><br>Patient meet all 4 criteria:<br><br>1. HAI criterion<br>2. Organism recovered from 1 or more blood specimens<br>3. Has HABSI within 72 hours<br>4. Blood isolates are different from those collected within 72 hours (different organism

	or different antibiogram[h])
**Persistent HABSI criterion**	Patient meet all 4 criteria:<br>1. HAI criterion<br>2. Organism recovered from 1 or more blood specimen<br>3. Has HABSI within 15 days<br>4. Blood isolates collected within 15 days are the same<br>5. Not fulfill “signs of recovery”[i]

[a]Described in Multimedia Appendix 2.
[b]Common skin commensal include *Corynebacterium* spp., *Bacillus* spp. (other than *Bacillus anthracis*), *Propionibacterium* spp., coagulase-negative staphylococci, viridians streptococci, *Streptococcus* spp. (other than *Streptococcus pneumonia*), *Aerococcus* spp., *Micrococcus* spp.
[c]Symptoms or signs of BSI for patients >1 year of age include fever (>38°C), chills, or hypotension, which is defined as the presence of a systolic blood pressure of less than 90 mmHg; for patients ≤1 year of age, these are described in Multimedia Appendix 3.
[d]Symptoms or signs of BSI for patients >1 year of age include fever (>38°C), chills, or hypotension, which is defined as the presence of a systolic blood pressure of less than 90 mmHg or a reduction of more than 40 mmHg from baseline in the absence of other causes of hypotension; for patients ≤1 year of age, these are described in Multimedia Appendix 3.
[e]Diagnosis of sepsis: ICD-9-CM is 003.1, 022.3, 038.0, 038.10, 038.11, 038.19, 038.2, 038.3, 038.40, 038.41, 038.42, 038.43, 038.44, 038.49, 038.8, 038.9, or 054.5.
[f]Secondary bloodstream infection is collected and classified for 3 reasons: first, for comparison with historical data; second, in order not to underestimate the burden of bloodstream infection; third, to identify the niche for further improvement.
[g]The detection rules did not adopt the criteria “organism recovered from blood also recovered from a nonblood cultures during the entire length of stay” due to 2 reasons: first, there is a lot of debate during consensus meeting; second, computer algorithm becomes very complicated.
[h]Antibiogram is reported as susceptible, intermediate, or resistance for each antimicrobial agent evaluated based on CLSI criteria. Different antibiograms are defined if in vitro susceptibility testing for 2 or more drugs is different, such as change from “susceptible” to “resistant or intermediate” or “resistant or intermediate” to “susceptible”.
[i]Signs of recovery: Fever subsides (body temperature resume to baseline) and lasts for more than 2 days.
