# Supplementary material for: A Web-Based, Hospital-Wide Health Care-Associated Bloodstream Infection Surveillance and Classification System: Development and Evaluation
Source: JMIR Med Inform. 2015 Sep 21;3(3):e31. doi: 10.2196/medinform.4171 (PMC4705006; doi:10.2196/medinform.4171)
Supplement: Multimedia Appendix 2 [file medinform_v3i3e31_app2.pdf]

Order name
**Central lines**
Cardiac catheter, both side
Cardiac catheter, one side
Percutaneous transluminal angiography
Stenting for head & neck vessel (one vessel)
Central venous catheter
Dual lumen subclavian catheter
Hickman catheter
Intraaortic balloon pumping
Jugular bulb catheteration
Percutaneous IV catheterization
Port-A catheter
Pulmonary artery catheterization
Swan-Gang catheterization
Therapeutic catheter implantation
Umbilical artery catheterization
Umbilical vein catheterization
**Intravascular catheters, devices or procedures***
A-V fistula needle set
Avf set for hemodialysis
Blood line
Cartridge blood tubing
Continuous ambulatory peritoneal dialysis
Continuous arteriovenous hemofiltration
Continuous venovenous hemodialysis
Continuous venovenous hemofiltration
Emergency hemodialysis
Filtryzer hollow fiber
Hemodialysis
Hemodialysis+ Erythropoietin
Hemoperfusion
Hickman hemodialysis catheter
Hollow fiber dialyzer
Quinton permcath dual lumen catheter
Apheresis leukocyte
Apheresis platelets
Blood transfusion
Cryoprecipitate
Deglycerolized red blood cells
Fresh frozen plasma
Frozen plasma
Leucocyte-poor red blood cells
Packed red blood cells
Plasma exchange

Plasmaphoresis
Removal of plasma from bone marrow
Washed red blood cells
White blood cells concentrate
Whole blood

* Several procedures are included in order to compensate the potential defect of data obtained from billing system.
