# Supplementary material for: A Web-Based, Hospital-Wide Health Care-Associated Bloodstream Infection Surveillance and Classification System: Development and Evaluation
Source: JMIR Med Inform. 2015 Sep 21;3(3):e31. doi: 10.2196/medinform.4171 (PMC4705006; doi:10.2196/medinform.4171)
Supplement: Multimedia Appendix 3 [file medinform_v3i3e31_app3.pdf]

**Age**	**Heart rate/ min**	**Breath rate/ min**	**Body temperature ( )**
Premature children	100	<40	38 or 36
<3 Months	90	<35	38 or 36
3~6 Months	80	<30	38 or 36
6~12 Months	70	<25	38 or 36
