# Supplementary material for: A Web-Based, Hospital-Wide Health Care-Associated Bloodstream Infection Surveillance and Classification System: Development and Evaluation
Source: JMIR Med Inform. 2015 Sep 21;3(3):e31. doi: 10.2196/medinform.4171 (PMC4705006; doi:10.2196/medinform.4171)
Supplement: Multimedia Appendix 4 [file medinform_v3i3e31_app4.pdf]

Appendix 4. Summary of characteristics and performances of computer-assisted healthcare-associated infection surveillance systems in the literatures.

**Author [reference]**	**Year**	**Target**	**Healthcare setting and patient population**	**Type of Rules**	**Results**
Du et al. [1]	2014	HAI	A 3500-bed Chinese tertiary hospital	Modified CDC based	Sen 98%, Spe 93%,
de Bruin et al. [2]	2013	BSI, PN, UTI, CRI	ICU in Vienna General Hospital, a 2133-bed tertiary care and teaching hospital	HELICS based	Sen 87%, Spe 99%, PPV 96%, NPV 95%.
van Mourik et al. [3]	2012	Drain-Related Meningitis	University Medical Center Utrecht, a 1042-bed tertiary medical center	Multivariable prediction model	AUC 0.963
Choudhuri et al. [4]	2011	Catheter related UTI	413-bed university-affiliated urban teaching hospital	CDC based	Sen 86.4%, Spe 93.8%, NPV 94.4%, k 0.80
Woeltje et al. [5]	2011	CLSBSI	4 non-ICU wards at Barnes-Jewish Hospital	Intersection of rules	Sen 95.2%, Spe 97.5%, PPV 90%, NPV 99.2%, k 0.908
McBryde et al. [6]	2009	BSI	ICU in 6 selected hospitals	CDC based	CLABSI, PPV 59%. Non-CLABSI, NPV 73%, Sen 35%, Spe 87%
Woeltje al. [7]	2008	CLSBSI	Barnes-Jewish Hospital, a 1,250-bed tertiary care academic hospital in Saint Louis, Missouri.	Intersection of rules	NPV 99.4%, Spe 44.2%
Pokorny et al. [8]	2006	HAI	The ICU in a 250-bed general hospital in Barcelona, Spain.	Intersection of rules	Sen 94.3%, Spe 83.6%, PPV 55.9%, NPV 98.5%
Leth et al. [9]	2005	sepsis	A Danish hospital	Intersection of rules	All 100%

Trick et al. [10]	2004	BSI	Cook County Hospital, a 600-bed public teaching hospital, and Provident Hospital, a 120-bed community hospital	Intersection of rules	Kappa 0.73
Bouam et al. [11]	2003	UTI, BSI, CRI	A 906-bed, tertiary-care teaching hospital	CDC based	Sen 91%, Spe 91%, PPV 88%, NPV 93%, Kappa 0.81

Abbreviation: BSI: Bloodstream infection, PN: pneumonia, UTI: Urinary tract infection, CRI: catheter-related infection, HAI: healthcare-associated infection, CLSBSI: Central line-associated BSI, CDC: Centers for Disease Control and Prevention, HELICS: Hospitals in Europe for Infection Control through Surveillance, Sen: sensitivity = true positive/(true positive +false negative), Spe: specificity = true negative /(true negative +false positive), PPV: positive predictive value = true positive/(true positive +false positive), NPV: negative predictive value = true negative /(true negative +false negative), AUC: Area under ROC curve.

1. Du M, Xing Y, Suo J, et al. Real-time automatic hospital-wide surveillance of nosocomial infections and outbreaks in a large Chinese tertiary hospital. BMC Med Inform Decis Mak **2014**; 14: 9.
2. de Bruin JS, Adlassnig KP, Blacky A, Mandl H, Fehre K, Koller W. Effectiveness of an automated surveillance system for intensive care unit-acquired infections. J Am Med Inform Assoc **2012**.
3. van Mourik MSM, Moons KGM, van Solinge WW, et al. Automated Detection of Healthcare Associated Infections: External Validation and Updating of a Model for Surveillance of Drain-Related Meningitis. PLoS ONE **2012**; 7(12): e51509.
4. Choudhuri JA, Pergamit RF, Chan JD, et al. An electronic catheter-associated urinary tract infection surveillance tool. Infect Control Hosp Epidemiol **2011**; 32(8): 757-62.
5. Woeltje KF, McMullen KM, Butler AM, Goris AJ, Doherty JA. Electronic surveillance for healthcare-associated central line-associated bloodstream infections outside the intensive care unit. Infect Control Hosp Epidemiol **2011**; 32(11): 1086-90.
6. McBryde ES, Brett J, Russo PL, Worth LJ, Bull AL, Richards MJ. Validation of statewide surveillance system data on central line-associated bloodstream infection in intensive care units in Australia. Infect Control Hosp Epidemiol **2009**; 30(11): 1045-9.
7. Woeltje KF, Butler AM, Goris AJ, et al. Automated surveillance for central line-associated bloodstream infection in intensive care units. Infect Control Hosp Epidemiol **2008**; 29(9): 842-6.
8. Pokorny L, Rovira A, Martin-Baranera M, Gimeno C, Alonso-Tarres C, Vilarasau J. Automatic detection of patients with nosocomial infection by a computer-based surveillance system: a validation study in a general hospital. Infect Control Hosp Epidemiol **2006**; 27(5): 500-3.
9. Leth RA, Moller JK. Surveillance of hospital-acquired infections based on electronic hospital registries. J Hosp Infect **2006**; 62(1): 71-9.
10. Trick WE, Zagorski BM, Tokars JI, et al. Computer algorithms to detect bloodstream infections. Emerg Infect Dis **2004**; 10(9): 1612-20.
11. Bouam S, Girou E, Brun-Buisson C, Karadimas H, Lepage E. An intranet-based automated system for the surveillance of nosocomial infections: prospective validation compared with physicians' self-reports. Infect Control Hosp Epidemiol **2003**; 24(1): 51-5.
