# Supplementary material for: A Web-Based, Hospital-Wide Health Care-Associated Bloodstream Infection Surveillance and Classification System: Development and Evaluation
Source: JMIR Med Inform. 2015 Sep 21;3(3):e31. doi: 10.2196/medinform.4171 (PMC4705006; doi:10.2196/medinform.4171)
Supplement: Multimedia Appendix 5 [file medinform_v3i3e31_app5.pdf]

10008440

Conformed HAI cases

已經判定的感染卡

Demographic Information

Name<br>姓名：吳ＯＯ	Account No.<br>帳號：11T[illegible]602	Chart No.<br>病歷號：5[illegible]2	Birthday<br>生日：1973[illegible]	Gender<br>性別：F

Infection unit 感染單位：T0-(-01 | Transfer date (in) 轉進日：2011/07/19 20:05:51 | Transfer date (out) 轉出日：2011/08/13 09:22:12 | 就診日：

Admission date 入院日：2011/07/14 | Discharge date 出院日：2011/08/13 | Discharge status 出院狀況：死亡 | TQIP: 是 | NNIS: 是

Infection date 感染判定日期：2011/08/01 | Type of HAI 主部位：BSI血流感染 | Subtype of HAI 次部位：PRIM- | 隔離：標準防護 (Isolation strategy)

Diagnosis

☑	譯	診斷日期 Diagnosis date	診斷名稱 Diagnosis description

Key In

Organism Identification

☑	送檢日期 Login date	(LogNo)檢體	檢查醫令 Lab order name	檢查結果 Lab test result	菌數 Number of colonies
☑	1000802	(0000087)BLOOD SITECVP	Blood Culture & Sensitivity	Enterococcus faecium	
	P:S AM:S TE:R VA:S CIP:R GMh:S TEC:S				

☐ No culture ☐ No pathogen

Key In

Prescription

☑	抗	用藥(起)	用藥(迄) Start and end date	劑量途徑 Dose and route	學名 Generic name	商品名 Brand name

Key In

Operation

☑	主	刀房 Operation room	手術醫令 vs 參與者 Operation orders and participants

Key In

Catheter Record

☑	醫令名稱 Order name	使用起迄日 Start and end date	留置部位 Site	執行人員 Executor

Key In

Predisposing factors

☐ DM ☐ Bile ☐ Ca ☐ WBC<1000 ☐ H/D ☐ P/D ☐ IV ☐ CVP ☐ D-L ☐ PICC ☐ Perm cath ☐ Port-A

☐ OTHER ☐ Organ Tx ☐ SWI ☐ UTI ☐ RTI ☐ GII ☐ Skin ☐ Ascites ☐ discharge ☐ urine ☐ sputum

備註 Comment Records

8/1 22:00 BT:38.2 8/5 D/L及CVP:no pathogen

儲存修改 Save | 取消修改 Cancel | 刪除卡片 Delete
